# CHILDBEARING STATUS, PARITY, AND COGNITIVE HEALTH AT MIDLIFE: A LONGITUDINAL APPROACH

**DOI:** 10.1093/geroni/igae098.2648

**Published:** 2024-12-31

**Authors:** Mieke Thomeer, Joseph Wolfe, Rin Reczek, Dee Ferguson, Rui Cao

**Affiliations:** University of Alabama at Birmingham, Birmingham, Alabama, United States; University of Alabama at Birmingham, Birmingham, Alabama, United States; The Ohio State University, Columbus, Ohio, United States; University of Alabama at Birmingham, Birmingham, Alabama, United States; The Ohio State University, Columbus, Ohio, United States

## Abstract

Childbearing histories matter for multiple health outcomes across the life course. Further, there is growing evidence that childbearing may also shape mid- and later-life cognitive health, although previous research is limited due to difficulty in accounting for the role of selection. Multiple factors that place women at risk for specific childbearing experiences also place them at risk for poor cognitive health—including socioeconomic background, health issues in early life, and cognitive ability in adolescence. We advance research by analyzing the 1979 National Longitudinal Survey of Youth (NLSY79) (N=2,257), a longitudinal nationally representative dataset from the US that began collecting data when women were in their late teens and early 20s. Final models account for a large array of social, economic, demographic, cognition, and health variables to estimate how childbearing histories matter for three cognitive health outcomes at age 48. In unadjusted models, we find that childbearing status, parity, and age at first birth are associated with self-reported memory and word recall, and age at first birth is also associated with backwards number counting. In fully adjusted and matched models, the only significant differences relate to memory. In future analysis, we will examine which specific factors drive associations, incorporate cognitive health at age 60, and replicate analyses in additional datasets. We demonstrate the role of childbearing as a determinant of cognitive health, suggesting the need to incorporate childbearing into life course models of dementia risk. We also draw attention to the importance of considering selection when identifying social determinants of cognitive health.

